# Graft Incorporation and Cup Migration in Acetabular Impaction Bone Grafting for Revision Hip Arthroplasty: A Systematic Review and Meta-Analysis of 1093 Hips

**DOI:** 10.1016/j.artd.2025.101929

**Published:** 2026-01-14

**Authors:** Artsiom Klimko, Octavian Andronic, Victor Yan Zhe Lu, Dominik Kaiser, Dimitris Dimitriou, Armando Hoch, Patrick O. Zingg

**Affiliations:** a Balgrist University Hospital, University of Zurich, Zurich, Switzerland; b Department of Orthopaedics and Traumatology, The Chinese University of Hong Kong, Hong Kong, China

**Keywords:** Femoral, Impaction bone grafting, Revision, Hip arthroplasty, Graft, Incorporation, Cup migration

## Abstract

**Background:**

Acetabular impaction bone grafting (IBG) is used to address bone loss in revision total hip arthroplasty (rTHA). We evaluated graft incorporation and cup migration after acetabular IBG in rTHA.

**Methods:**

Systematic search of MEDLINE, EMBASE, and Scopus from inception to June 30, 2024 (PROSPERO CRD42024557047). Studies of acetabular IBG in rTHA with ≥12-month follow-up were included. Outcomes were graft incorporation and horizontal (i.e., lateral to medial axis) and vertical cup migration. Prespecified subgroup analyses assessed bone-loss severity, graft type, additional fixation, and age. Random-effects meta-analyses were used; heterogeneity was quantified with I^2^. Risk of bias was assessed with the Methodological Index for Non-Randomized Studies.

**Results:**

Nineteen studies (1093 hips) were included; weighted follow-up was 8.0 years (range 2.0-16.9). Pooled graft incorporation was 89% (95% CI [confidence interval] 79-96; I^2^ 85%). Mean lateral migration was 2.4 mm (95% CI 0.53-4.27) and mean superior migration 4.2 mm (95% CI 1.61-6.75); heterogeneity was high (I^2^ 100% for both). Lateral migration was greater in cohorts with ≥5 years’ follow-up than <5 years (3.65 vs 1.16 mm; *P* = .018). No significant differences in incorporation were detected by bone-loss severity, graft type, or age; however, subgroup analyses were frequently underpowered and exploratory.

**Conclusions:**

Acetabular IBG can achieve mid- to long-term graft incorporation of 89%, but estimates are imprecise due to heterogeneous (I^2^ = 85%), predominantly retrospective evidence. Migration occurs—most commonly cranial—and may accrue over time; values often remain within commonly accepted surveillance thresholds but warrant structured radiographic follow-up. Given high heterogeneity, variable outcome definitions, and potential small-study/publication bias, recommendations should be interpreted cautiously.

## Introduction and background

Acetabular bone loss presents a considerable challenge in revision total hip arthroplasty (rTHA), often jeopardizing initial fixation and long-term stability. Particularly in younger and more active patients, restoring bone stock is essential to accommodate potentially necessary further revisions. Impaction bone grafting (IBG) has gained traction as a reliable method to increase deficient acetabular bone stock, offering both mechanical support and biological restoration of host bone [1,2].

Early evidence by Slooff et al [3]. introduced IBG as an effective approach for addressing protrusion defects of the acetabulum. Subsequent work by Schreurs et al [2] [4]. confirmed these findings, demonstrating high implant survival rates—exceeding 90% at 10 to 20 years—when IBG was paired with cemented acetabular components. These pioneering studies underscored the technique’s capacity to restore structural support, promote graft incorporation, and achieve durable fixation, even in cases presenting substantial bone loss.

Nevertheless, the degree of graft incorporation and magnitude of cup migration vary across different patient populations and surgical protocols [5,6]. Notably, excessive cup migration, whether vertical or horizontal, has been linked to aseptic loosening and eventual implant failure [7]. In contrast, stable fixation with minimal implant displacement often corresponds to favorable clinical results [8]. Although the benefits of IBG with uncemented components have been reported, [9] there is ongoing debate on whether uncemented cups necessitate greater native bone contact than cemented implants for optimal stability [10,11]. Previous experience from femoral IBG studies suggest great potential in terms of graft incorporation and stability [12, 13, 14, 15].

Owing to the limited consensus on best practices—such as the use of graft type, fixation devices, or specific operative strategies—this systematic review and meta-analysis seeks to examine current evidence regarding graft incorporation and cup migration in acetabular IBG for rTHA. Understanding these outcomes may guide surgeons toward more standardized approaches, improved patient selection, and ultimately enhance the longevity of acetabular reconstruction in the revision setting.

## Material and methods

### Systematic search strategy

This systematic review was performed according to the Preferred Reporting Items for Systematic Reviews and Meta-Analyses guidelines [16] and is registered in the International Prospective Register for Systematic Reviews (registration number: CRD42024557047). The review includes studies reporting on outcomes following acetabular IBG in rTHA, with emphasis on survivorship, graft incorporation, and cup migration. A comprehensive literature search was conducted in 3 electronic databases (MEDLINE (via PubMed), EMBASE, and Scopus) from their respective inceptions to June 30, 2024. The search terms included combinations such as “impaction bone grafting,” “acetabular reconstruction,” “revision hip arthroplasty,” “cup migration,” “graft incorporation,” and “construct survivorship.” Only studies in English and involving human participants with at least 12 months of follow-up were considered. Additional publications were identified by screening reference lists of key articles. Duplicate records were removed prior to screening.

### Selection process and data extraction

Two independent reviewers (A.K. and V.L.) screened article titles and abstracts, followed by full-text assessment of potentially eligible studies. In case of disagreement between the reviewers, a third author (O.A.) was consulted to reach a consensus. Studies were included if they were peer-reviewed, in English, had a minimum level of evidence of IV according to the Oxford Centre for Evidence-Based Medicine (2011), reported outcomes of acetabular IBG in rTHA, and featured at least 12 months of follow-up. Publications such as reviews, technique descriptions, or editorials, as well as cadaveric or animal investigations and case series with fewer than 10 hips, were excluded. Primary total hip arthroplasty studies were also excluded, as the aim was to focus solely on revision procedures. Extracted data from each included study encompassed the number of hips, patient demographics, follow-up durations, bone loss stratification, graft types, bone chip size, fixation strategies, and the principal outcomes of cup migration, graft incorporation. Horizontal cup migration was evaluated to look for medial or lateral displacement of the acetabular component.

### Risk of bias assessment

The Methodological Index for Non-Randomized Studies (MINORS) was used to evaluate the risk of bias in each study [17]. This instrument allocates scores based on the comprehensiveness and clarity of reporting for key methodological domains: (1) clearly stated aim; (2) inclusion of consecutive patients; (3) prospective data collection; (4) endpoints appropriate to the aim; (5) unbiased assessment of endpoints; (6) follow-up period appropriate to the aim; (7) loss to follow-up <5%; (8) prospective calculation of study size. For comparative studies, 4 additional items are scored (maximum 24 points): (9) adequate control group; (10) contemporary groups; (11) baseline equivalence of groups; (12) adequate statistical analyses. Items were scored 0/1/2, corresponding to (“not reported” / “reported but inadequate” / “reported and adequate”).

Two reviewers (A.K., V.L.) conducted independent MINORS scoring after a pilot calibration on a random subset of studies to harmonize item interpretation. Disagreements were resolved by consensus, with senior adjudication (O.A.) when needed. Noncomparative studies were scored out of 16 points, while comparative ones out of 24 – in Table 1, we report raw scores (out of 16 or 24, as appropriate).

**Table 1:** Overview of included studies.

Author and year	Numbers of hips in study	Mean age (y)	Calculated MINORS score	Gender - Male/Female	Mean follow-up (range)	Indications for revision	Bone graft type and chip size	Additional fixation	Severity of bone loss	Cemented/Uncemented cup number	Reason for re-revision failure (number of hips)
Hak Lian Teh et al., 2023 [18]	38	55 (21-81)	13/16	Not specified/Not specified	6	Avascular necrosis of femoral head (22 patients), Revision surgery due to aseptic loosening (9 patients), etc.	Autografts (10 hips), Frozen irradiated femoral head allografts; 5-10 mm	M/S	Paprosky classification: Type 2A: 8, Type 2B: 12, etc.	0/38	Aseptic loosening (2 Paprosky grade 3A, 1 Paprosky 3B)
Chao Yang et al. 2023 [19]	29	59.3 (29-81)	12/16	50/50	9.4 (2.4-14.0)	Aseptic loosening: 28 (96.6%), Periprosthetic fracture: 1 (3.4%)	Irradiated fresh-frozen femoral head allograft; 0.5-1.0 cm^3^	M/S	Paprosky classification: Type IIB: 4, Type IIIC: 3, etc.	29	Aseptic loosening (1), infection (1)
Quarto et al., 2021 [20]	40	71.4 (33-93)	15/16	20/80	14.3 (10-22)	87.5% aseptic mobilizations of the acetabular component, 7.5% chronic dislocations	Fresh-frozen femoral head allograft; 0.5-1 cm^3^	R/C, M/S	Paprosky classification: Type IIIA: 27, Type IIIB: 13	40	Aseptic failure (1), septic failure (1)
Stigbrand et al., 2020 [21]	17	73 (49-87)	14/16	53/47	2	Acetabular bone loss	Not specified	R/C	Combined segmental and cavitary defects: 6 (35.3%)	17	Mechanical failure with proximal migration of 6 mm
Zhang et al., 2020 [22]	18	67.5 (65.3-69.0)	13/16	44.4/55.6	5.1 (4.67-5.48)	Aseptic loosening	Fresh-frozen femoral head allograft	M/S	Paprosky classification: Type IIIA: 11, Type IIIB: 7	Not specified	None reported
Gerhardt et al., 2018 [23]	20	70 (61-79)	14/16	55/45	2	Cup loosening with osteolytic defects (14), revision for infection (3)	Fresh-frozen femoral head allograft; 7-10 mm	None	AAOS classification: Type II	20	Recurrent dislocations
Green et al., 2018 [24]	123	64.3 (26-97)	12/16	50/50	16.9 (14.7-24.9)	Aseptic loosening	Irradiated allograft bone; 0.5-1.0 cm^3^	M/S	AAOS classification: Type I: 27, Type II: 63, etc.	123	Infection (10), aseptic loosening (8), dislocation (3), etc.
Fadulelmola et al., 2017 [25]	80	65.6 (35-84)	14/16	41.9/58.1	6.5 (1-13)	Not specified	Fresh frozen femoral heads with cartilage retained; ∼8 mm	None	AAOS classification: Type II: 68, Type III: 6	80	Socket migration (1), recurrent dislocations (2)
Hosny et al., 2018 [6]	26	71 (49-91)	12/16	44/56	4.1 (2.5-6.5)	Aseptic loosening in 19 hips, combined instability and AL in 2 hips, infection in 3 hips	Fresh-frozen femoral head allograft	R/C	Paprosky classification: Type IIB: 2, Type IIC: 4, etc.	26	None
Rowan et al., 2016 [26]	36 (AIBG), 17 (TM)	68 (AIBG) / 74 (TM)	15/16	AIBG: 41.2 (14/34), TM: 73.3/26.7	AIBG: 5.9 / TM: 5.4 (AIBG: 0.7-12.0 / TM: 0.8-10.4)	Osteolysis, Instability, Sepsis, Periprosthetic fracture (femur)	AIBG: Fresh-frozen nonirradiated allograft	M/S, TM	AIBG: Paprosky grade 2C (27%), TM: Paprosky grade 3A (41%)	Not specified	AIBG: Cup spin-out, sepsis; TM: None
García-Rey et al., 2015 [27]	204	69 (Not specified)	10/16	30/70	10 (5-17)	Severe bone defects (Paprosky 3A and 3B)	Fresh-frozen femoral head allograft; 0.7-1 cm^3^	M/S	Paprosky classification: Grade 3A: 100 (49.0%), Grade 3B: 104 (51.0%)	177/27	Recurrent dislocation (3), infection (2), cup loosening (8), etc.
Schreurs et al., 2015 [2]	11	67.5 (43-83)	15/16	30/70	10 / 28 (5-15 / 26-30)	Aseptic loosening, wear, mismatch during femoral revision	Allograft bone chips; 8-12 mm	M/S	Combined cavitary and segmental defects	11	Aseptic loosening at 12 years (1), malpositioning (1)
Bilgen et al., 2012 [28]	15	52.1 (36-73)	12/16	33.3/66.7	8.1 (4.8-10.8)	Aseptic loosening: 9 (60%), Septic loosening: 6 (40%)	Fresh-frozen femoral head allograft; 4-10 mm	M/S	Paprosky classification: Type 2B: 1, Type 2C: 2, etc.	Not specified	Infection (1), nonincorporation (1)
Mehendale et al., 2009 [11]	50	64 (Not specified)	12/16	35/65	3.75 (3.75)	Aseptic revision with acetabular bone stock loss	Irradiated femoral head allograft; 5 mm	None	Paprosky classification: Type 1A: 2, Type 2B: 15, etc.	44/6	Aseptic loosening (5), infection (1)
Liu et al., 2008 [29]	15	48.2 (36-73)	17/24	66.7/33.3	4.3 (2-7)	Post-traumatic arthritis and bone loss after acetabular fractures	Autogeneic particulate cancellous bone; 1 mm^3^	M/S	American Orthopaedic Association classification: Type I: 3, etc.	10/5.0	None reported
Ochs et al., 2008 [30]	79	72.5 (Group A) / 69.9 (Group B) (47-90 (Group A) / 46-83 (Group B))	10/16	Group A: 40 (16/40), Group B: 42.1 (16/38)/Group A: 60 (24/40), Group B: 57.9 (22/38)	Group A: 3.15 / Group B: 2.05 (Group A: 1.92-4.25 / Group B: 1.17-2.92)	Aseptic cup loosening, aseptic cup and stem loosening	Group A: Frozen nonirradiated allograft, Group B: Freeze-dried irradiated allograft vitalized with autologous marrow; 1 cm^3^	R/C	American Academy of Orthopaedic Surgeons classification: Type I: 8 (10.1%), Type II: 2 (2.5%), Type III: 69 (87.3%)	79	None
van Haaren et al., 2007 [31]	71	69.1 (32.8-91.4)	9/16	Not reported/Not reported	7.2 (1.6-9.7)	Aseptic loosening (59), septic loosening (12)	Fresh-frozen femoral head allograft; 0.7-1 cm	M/S	AAOS classification: Type I: 13 (18.3%), Type II: 17 (23.9%), Type III: 35 (49.3%), Type IV: 6 (8.5%)	71	Aseptic loosening (20)
Buckley et al., 2005 [10]	123	64.3 (26-97)	14/16	Not reported/Not reported	5 (2-12)	Aseptic loosening	Irradiated fresh-frozen femoral head allograft; 0.5-1 cm	R/C	AAOS classification: Grade I: 27 (22.0%), Grade II: 63 (51.2%), Grade III: 28 (22.8%), Pelvic dissociation: 5 (4.1%)	123	Deep sepsis (8), persistent early dislocation (2), aseptic loosening (3)
Pitto et al., 1998 [32]	81	60 (Not specified)	10/16	Not specified/Not specified	6.5 (3-9)	Aseptic loosening	Autologous and homologous bone grafting with structural bone blocks; 0.5 cm^3^	R/C	D'Antonio classification: Type I, II, III, IV	81	Recurrent dislocation of the femoral head

TM, trabecular metal; AAOS, American Academy of Orthopaedic Surgeons; AL, aseptic loosening; AIBG, acetabular impaction bone grafting.

The included studies were predominantly retrospective, with MINORS scores ranging from 9 to 17. Most were classified as level III or IV evidence, and no randomized controlled trials were identified. Common methodological limitations included incomplete follow-up data and a lack of comparator groups (Table 1).

### Exploratory meta-analyses

Where comparable data were available, meta-analyses were conducted on the following outcomes: construct survivorship, graft incorporation, and cup migration rates. These analyses were carried out using the 'metafor' package in R (R Core Team, R Foundation for Statistical Computing, Vienna, Austria). A random-effects model with inverse variance weighting was employed to pool effect sizes, given the expected variability among the included studies. For continuous outcomes, standardized mean differences were calculated, while for binary outcomes, such as complications or the need for re-revision, pooled proportions were computed [33].

Heterogeneity was assessed using the I^2^ statistic and Cochran’s Q test, with prediction intervals also calculated to account for the expected variability in future studies. Heterogeneity was classified as moderate if I^2^ exceeded 40% and high if it exceeded 75% [34]. When summary statistics were reported as medians and ranges but no means or standard deviations were provided, means and standard deviations were estimated using recognized methods, including the Wan et al. estimator, to approximate normal distributions and allow consistent pooling of data [35].

### Subgroup analysis

Subgroup analysis examined potential influences of different factors on graft incorporation and cup migration, including the severity of bone loss (Endoklinik or Paprosky I + II vs III + IV), graft type (fresh-frozen, irradiated, or autograft) and size (chips, bulk or other), additional fixation techniques (rings, cages, or screws) and patient age. We classified nonstructural grafts as morselized cancellous chips (typically ≤10–12 mm), and structural grafts as bulk corticocancellous blocks/segments used to bridge segmental loss or pelvic discontinuity; autograft denotes patient-derived bone and allograft denotes donor bone (fresh-frozen or irradiated).

Subgroup analyses were included only if there were at least 4 studies in total to ensure robust statistical analysis, as has been done in a previous review [14] Age was dichotomized at 65 years, a threshold commonly used in hip arthroplasty literature and reflective of typical Medicare eligibility criteria [36, 37, 38]. This cutoff distinguishes younger, potentially more active patients from those over 65, who often have different bone quality and comorbidity profiles. We also planned subgroup analyses based on whether cups were cemented or uncemented. However, because only 76 hips across the included studies were managed with uncemented cups, the available data were insufficient for a robust comparison.

## Results

### Database search

**Figure 1:**
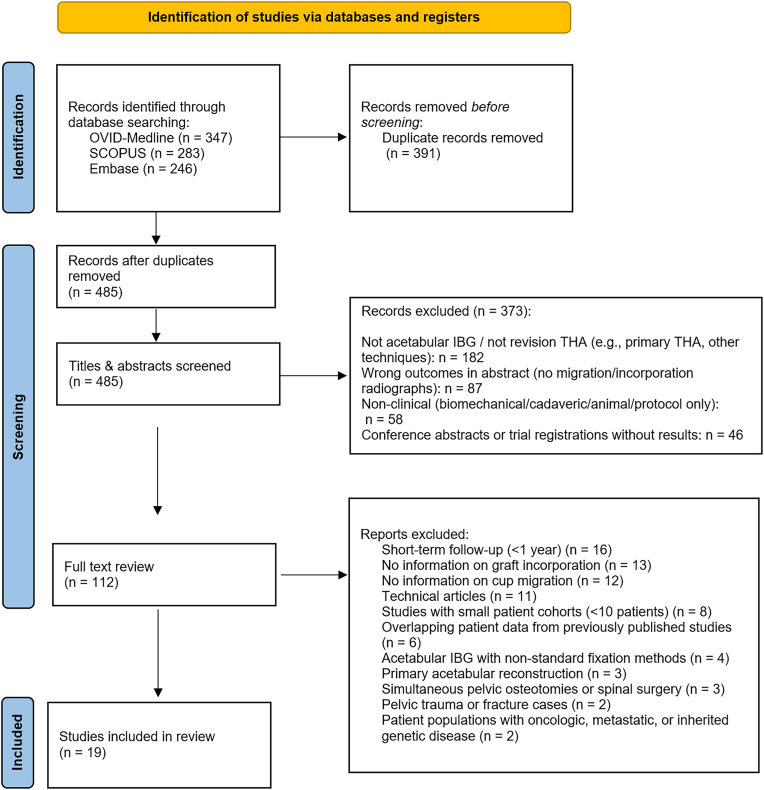
PRISMA flowchart for study selection into systematic review. PRISMA, Preferred Reporting Items for Systematic Reviews and Meta-Analyses; THA, total hip arthroplasty.

The systematic search yielded a total of 876 citations from 3 databases: OVID-Medline (347 records), SCOPUS (283 records), and Embase (246 records). After removing 391 duplicate records, 485 studies remained for further analysis. During the title and abstract screening phase, 373 studies were excluded based on predefined criteria. This left 112 studies for full-text review, of which 93 were excluded for reasons such as technical articles, primary acetabular reconstruction, use of nonstandard fixation methods, or lack of information on cup migration or graft incorporation. Ultimately, 19 studies met the inclusion criteria and were included in the systematic review and meta-analysis (Fig. 1).

### Demographics

A total of 1093 hips were reported across the 19 included studies. The number of hips per study ranged from 11 to 204, with a median of 50. The weighted mean age of patients across all studies was 65.9 years (range, 48.2 to 74), and female patients constituted approximately 59.9% of the overall cohort.

Outcome-specific denominators were not reported by all 19 studies—therefore, number of studies and hips differ by outcome. Lateral migration pooled 6 studies / 259 hips; superior migration pooled 8 studies / 332 hips; radiolucent lines summarized 11 studies / 642 hips; quantitative imaging subsets ranged from 2-3 studies / 38-104 hips as detailed below. Graft incorporation was reported by a subset of studies and is analyzed on that subset only.

The sample-size–weighted follow-up was 8.0 years (range, 2.0-16.9 years). Of the 13 studies (713 hips) that explicitly categorized indications for revision, 649 hips (91.0%) were revised for aseptic loosening, 27 (3.8%) for infection, 5 (0.7%) for recurrent dislocation, and 1 (0.14%) for periprosthetic fracture; the remaining 31 hips (4.3%) fell under other causes (eg, avascular necrosis, post-traumatic arthritis). Six studies (380 hips) did not provide a detailed breakdown of revision indications but generally cited severe bone loss or post-traumatic deformity.

Some studies specified exclusively cemented cups, whereas others used uncemented cups or a combination of both. Unfortunately, further analysis is precluded by significant disparities in incidence (cemented—931 hips vs uncemented—76 hips). Roughly two-thirds of reconstructions relied on “medium” morselized grafts in the 2–8 mm range, while the remainder were built with coarser fragments larger than 8 mm (Table 1). Only a handful of investigations deliberately chose at, or beyond, the 1-cm scale—most notably Li et al. (8–12 mm) [39], Schreurs et al. (8–12 mm) [2], Ochs et al. (∼1 cm^3^) [30], and Mehendale et al. (mixed 5 mm chips plus occasional 1 cm pieces) [11]. These outliers account for the right-skew in particle size distribution but were numerically insufficient to influence overall migration or incorporation trends. Only one study reported using structural bone blocks, when necessary, together with morselized bone [32].

Applying additional fixation devices such as screws, meshes, or cages, was frequent, particularly in cases with significant bone defects. Variables such as body mass index and whether the right or left hip was affected were reported inconsistently, limiting more detailed subgroup analyses for these factors. Furthermore, a direct comparison between cementless and cemented cups was not possible because only one study included cementless cups [18].

### Small-study publication bias effects

**Supplementary Figure 1:**
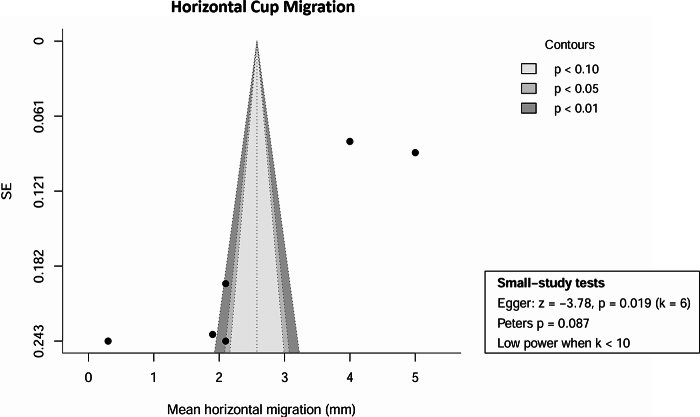


**Supplementary Figure 2:**
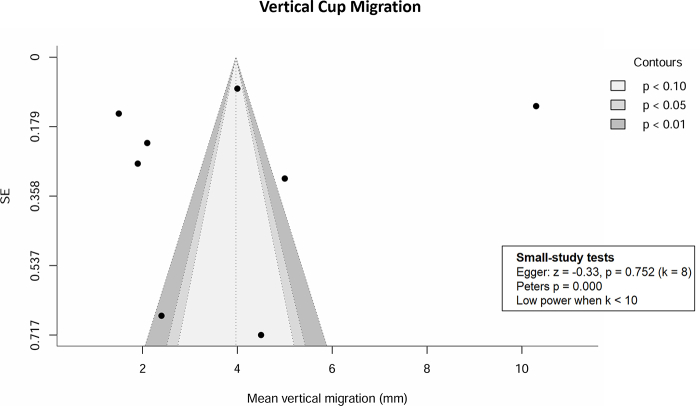


**Supplementary Figure 3:**
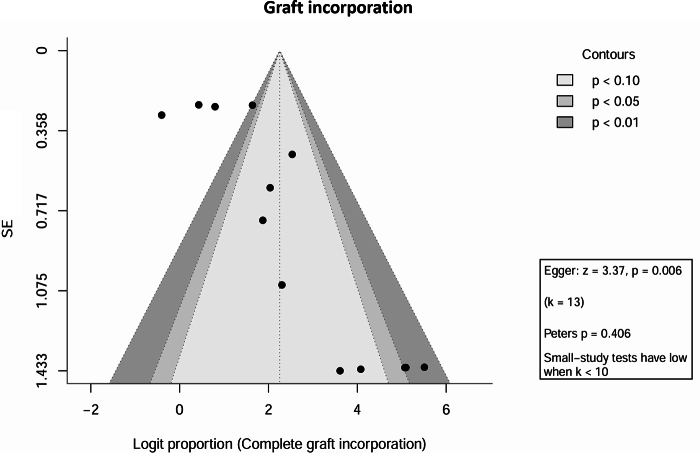


Contour-enhanced funnel plots and Egger’s tests are shown in Supplementary Figures S1–S3. Egger’s regression indicated asymmetry for graft incorporation (z = 3.37, *P* = .006; k = 13), consistent with smaller studies reporting higher incorporation. For vertical migration, Egger’s test was nonsignificant (z = −0.33, *P* = .752; k = 8), whereas Peters’ test was significant (*P* < .001), yielding mixed signals. For horizontal migration, Egger’s test suggested asymmetry (z = −3.78, *P* = .019; k = 6), with smaller studies tending toward lower migration. Given the small number of studies for migration outcomes (k < 10) and heterogeneity in reporting, these findings are exploratory and should be interpreted with caution.

### Meta-analysis

#### Cup migration

For clarity, horizontal migration is defined along the mediolateral axis (positive = lateral, negative = medial), and vertical migration along the superoinferior axis (positive = superior, negative = inferior). Across all included studies, no medial or inferior migrations were reported, confirming that cup micromotion was confined to lateral and superior directions, likely due to abduction pull-out.

**Figure 2:**
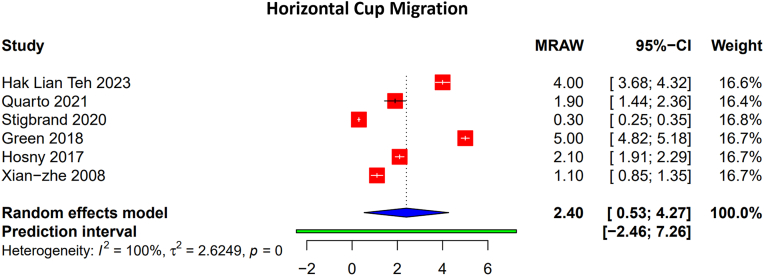
Pooled rates of horizontal cup migration. The forest plot shows the proportion of surviving grafts with the size of squares representing the weight of each study. I^2^ represents heterogeneity.

Data on lateral acetabular component migration were available from 6 studies encompassing 259 hips Table 2. The pooled mean lateral migration was 2.4 mm (95% CI: 0.53-4.27 mm) as shown in Figure 2 but significant heterogeneity was observed (I^2^ = 100%, *P* < .01). Individual mean values ranged from 0.3 mm to 5.0 mm.

**Table 2:** Cup migration.

Author and year	Numbers of hips in study	Methodology to evaluate graft incorporation	Cup migration - unspecific direction (mm)	Cup migration - vertical direction (mm)	Cup migration - lateral direction (mm)	Time point at which migration was evaluated (y)
Teh et al., 2023 [18]	38	Plain radiographs	NA	3-5	3-5	6
Yang et al., 2023 [19]	29	Plain radiographs	8, 10, 12	NA	NA	9.4 (2.4-14.0)
Quarto et al., 2021 [20]	40	Plain radiographs	NA	2.1 ± 1.4	1.9 ± 1.5	14.3
Stigbrand et al., 2020 [21]	17	CT-based micromotion analysis and volumetric QCT	NA	1.5 -0.6	0.3	0.12, 2
Gerhardt et al., 2018 [23]	20	Plain radiographs	NA	>5 (6, 8)	NA	NA
Green et al., 2018 [24]	123	Plain radiographs	NA	10.3	5	16.9 (14.7-24.9)
Hosny et al., 2018 [6]	26	Plain radiographs	NA	1.9	2.1	4.1 (2.5-6.5)
Rowan et al., 2016 [26]	36 (AIBG), 17 (TM)	Plain radiographs	NA	4.5 ± 4.3 (AIBG), 2.3 ± 2.1 (TM)	NA	NA
García-Rey et al., 2015 [27]	22-Jul	Plain radiographs	>5	NA	NA	10 (5-17)
Bilgen et al., 2012 [28]	15	Plain radiographs	>2 (1 case), <2 (10 cases)	NA	NA	8.1
Mehendale et al., 2009 [11]	50	Plain radiographs	Average 5.1 (2-25)	NA	NA	1
Liu et al., 2008 [29]	15	Plain radiographs	NA	2.2 (0-10), 2.4 (0-11)	1.1 (0-5), 2.1 (0-13)	7
van Haaren et al., 2007 [31]	71	Plain radiographs	>5	NA	NA	NA

TM, trabecular metal; QCT, quantitative computed tomography.

**Figure 3:**
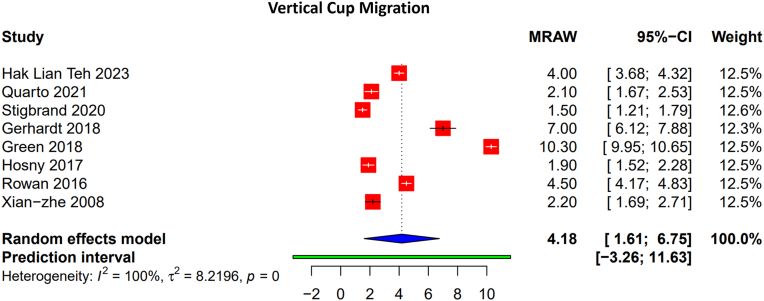
Pooled rates of vertical cup migration. The forest plot shows the proportion of surviving grafts with the size of squares representing the weight of each study. I^2^ represents heterogeneity.

Eight studies involving 332 hips provided measurements of superior cup migration, where the pooled mean superior migration was 4.2 mm (95% CI: 1.61-6.75 mm) (Fig. 3), and again heterogeneity was significant (I^2^ = 100%, *P* < .01). Reported superior migration means spanned from 1.5 to 10.3 mm, indicating considerable variability among cohorts.

#### Graft incorporation

**Figure 4:**
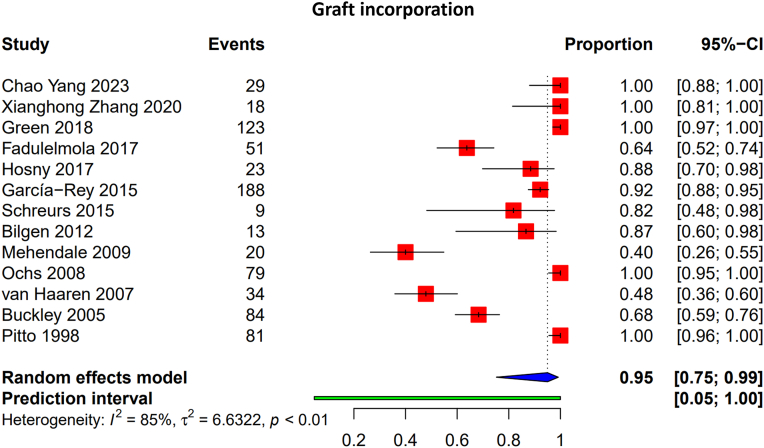
Pooled graft incorporation rates in acetabular impaction bone grafting. The forest plot shows the proportion of surviving grafts with the size of squares representing the weight of each study. I^2^ represents heterogeneity.

Thirteen studies with a total of 1093 hips reported on graft incorporation Table 3. The pooled rate of successful incorporation was 95% (95% CI: 75%-99%) (Fig. 4). Although heterogeneity remained high (I^2^ = 85%, *P* < .01), most studies demonstrated incorporation rates exceeding 80%.

**Table 3:** Graft incorporation statistics.

Author and year	Numbers of hips in study	Time point at which graft incorporation was evaluated (y)	Complete graft incorporation (n, %)	Partial graft incorporation (n, %)	Bridging cortex visible (earliest report)	≥50 % cortices continuous (timing / % hips)	First trabecular bridging (timing)	Full trabecular remodeling (timing / % hips)	Early thin radiolucent lines: n(%) hips / first seen (mo)
Yang et al. 2023 [19]	29	9.4 (2.4-14.0)	100%	NA	NA	NA	NA	12 mo-100 %	NA
Stigbrand et al., 2020 [21]	17	0.12, 2	NA	NA	6 wk	Not stated; density +14 % at 6 wk	≈3 mo	24 mo-23 % BMD gain (QCT)	1/17 (6 %) at 5-7 mo (zone I)- static
Zhang et al., 2020 [22]	18	1	100%	NA	NA	NA	NA	12 mo-100 % remodeling	NA
Gerhardt et al., 2018 [23]	20	0.25, 0.5, 1, 2	NA	NA	NA	NA	NA	24 mo-BMD +9 % (DXA)	NA
Green et al., 2018 [24]	123	16.9	100%	NA	NA	NA	NA	>24 mo-95 % osseointegration	4/57 (7 %) at 3-6 mo-static
Roessler et al., 2018 [40]	44	2.17	NA	NA	NA	NA	NA	Final FU-95 % osseointegration	3/44 (7 %) at 4-6 mo-static
Drampalos et al., 2017 [41]	42	9.3 (6-13)	NA	NA	NA	NA	NA	>24 mo-30/36 (83 %) good remodeling	NA
Fadulelmola et al., 2017 [25]	80	6.5 (2-13)	68.9%	27%	NA	NA	NA	Final FU-51/74 (69 %) complete	NA
Hosny et al., 2018 [6]	26	4.1 (2.5-6.5)	23 hips	NA	NA	NA	NA	NA	2/26 (8 %) at 6 mo-static
Arumugam et al. 2015 [42]	68	NA	92%	8%	NA	NA	NA	92 % at final FU	NA
Schreurs et al., 2015 [2]	11	10 / 28 (5-15 / 26-30)	87%	NA	NA	NA	NA	Re-revisions: 9/9 trabecular incorporation	NA
Bilgen et al., 2012 [28]	15	NA	86.6%	86.7%	NA	NA	NA	13/15 (87 %) complete at FU	1/15 (7 %) at 3-5 mo-static
Lee and Nam, 2011 [43]	71	2	NA	NA	NA	NA	NA	All cases showed new trabeculae	NA
Comba et al., 2009 [1]	30	7.2 (2.8-19)	NA	NA	6 wk	12 mo-> 50 % continuous; 24 mo-93 %	≈3 mo	>24 mo-28/30 (93 %) full	2/30 (7 %) at 4-7 mo-static
Mehendale et al., 2009 [11]	50	0.0082, 0.25, 0.5, 1+	40%	NA	NA	NA	≈4–6 mo	>24 mo-20/50 (40 %) full	8/50 (16 %) at 5-7 mo-static
Buttaro et al., 2008 [44]	23	2.98	NA	NA	NA	NA	NA	Histology: woven-bone by 6-12 mo	NA
Zhe et al. 2008 [29]	15	0.25, 0.5, 1, 1+	NA	NA	NA	NA	NA	Continuous trabeculae at FU	NA
Ochs et al., 2008 [30]	79	2.62 (1.17-4.25)	100%	NA	NA	NA	NA	12 mo-79/79 (100 %) full	3/79 (4 %) at 4-6 mo-static
Palm et al., 2007 [45]	87	9 (7-11)	NA	NA	8 wk	≈10 mo-> 50 % continuous	≈5 mo	>24 mo-“obvious” trabecular span in 41/87 (47 %)	6/87 (7 %) at 4-8 mo-static
van Haaren et al., 2007 [31]	71	7.2 (1.6-9.7)	NA	61%	NA	NA	Histology: woven by 10 mo	Radiology: 34/51 (66 %) complete	9/71 (13 %) at 6-8 mo-some progressed
Buckley et al., 2005 [10]	123	5	84%	NA	12 mo-75 % intact cortex	>24 mo-84 % trabecular	≈5-6 mo	Final FU-84 % full	5/123 (4 %) at 3-6 mo-static
Pitto et al., 1998 [32]	81	Within 1	NA	NA	3 mo-all grafts fused	NA	≈3 mo	NA	NA

TM, trabecular metal; FU, follow-up; BMD, bone mineral density; QCT, quantitative computed tomography.

#### Timing of cortical repair

Three studies (122 hips) reported when the first bridging cortex became visible, with a size-weighted mean of 4.4 months [45, 21, 22]. Further 9 studies (202 hips) specified when ≥50% of hips showed cortical continuity—a subset [6,10,28, 40, 31] noted an “intact cortical shell” or “bridging trabeculae” on their 1-year radiographs, while another subset [1,11,46] reported 84–93% continuity at 2–5 years, and even irradiated-graft cohorts [10,30] showed complete repair by final follow-up. The range for this ≥50% milestone spanned 6 to 12 months, with a weighted mean of 10.8 months, indicating that virtually all surviving impaction-graft reconstructions exhibit a continuous cortical rim within the first postoperative year.

#### Trabecular incorporation

Six of the studies (362 hips) detailed when radiographic trabecular bridging first became visible, yielding a size-weighted mean of 4.7 months (range, 3-6) [6,32,45, 21, 22, 28]. Eight cohorts (519 hips) quantified full trabecular remodeling at ≥ 24 months [6,10,11,30,32,45, 21, 22, 28]. The weighted average of trabecular incorporation amount was 81% (range, 66–100%). High rates were seen in both fresh-frozen and irradiated grafts—Buckley et al. achieved 84% of 123 hips [10] and Ochs et al. 100% of 79 hips [30]—whereas Mehendale’s et al [11]. irradiated series reached only 40%, indicating that while sterilization alone doesn’t impede trabecular consolidation with proper impaction, technical or defect-related factors can significantly undermine it.

#### Radiolucent lines

Interface lucencies were specifically tracked in 11 studies (642 hips) [1,6,10,11,30,45,21,28, 40, 31,24]. Thin (<2 mm) lucencies were usually seen between 3- and 8-months postoperative, almost always in DeLee–Charnley zones I–II. The size-weighted mean prevalence was 5.9% of hips and 5.1% of evaluated zones. None found a statistically significant link between early, thin, nonprogressive lines and later migration or re-revision. In every study, lines that eventually widened (>2 mm) or progressed round the socket were the ones that heralded aseptic failure [31]; static lines were clinically irrelevant.

#### Histology

Two papers (van Haaren et al., 13 core biopsies; Buttaro et al., 9 retrieval biopsies) examined the graft bed directly [31,44]. Specimens obtained within the first 12 postoperative weeks were entirely avascular; by 6 to 12 months, revascularization fronts and woven-bone deposition had replaced over 80% of the original graft volume, whereas all cups that failed aseptically (n = 9) retained broad islands of necrotic cancellous bone and interstitial fibrous tissue. In the late postoperative interval (>24 months), a subset of specimens—4 of 13 in van Haaren’s series—showed complete conversion to mature lamellar trabeculae, underscoring that full structural integration occurs by this time in successful reconstructions, while incomplete turnover remains predictive of aseptic failure.

#### Quantitative imaging

Two studies (38 hips) [23,47] evaluated graft integration by serial dual-energy X-ray absorptiometry), demonstrating a consistent trajectory of bone restitution. Across both series, graft bone mineral density increased steadily, with an average gain of 9% by 24 months and a particularly pronounced 14% rise in the cranial sector. Importantly, no transient demineralization dip was observed, indicating uninterrupted mineral incorporation into the impacted bone graft.

Two studies (55 hips) employed low-dose quantitative computed tomography to map volumetric density changes in cavitary or combined defects [30,21]. Stigbrand et al. reported a 14% density increase as early as 6 weeks postoperatively and a cumulative 23% gain at 2 years. Ochs et al. followed 38 γ-irradiated, freeze-dried allografts and measured Hounsfield units (HU) rising from approximately 480 immediately after implantation to 710 at 12 months, approaching adjacent native cancellous bone (720 HU).

Two studies (104 hips) [32,44] used 99mTc-MDP scintigraphy or single-photon emission computed tomography for functional assessment at 4 to 6 months. Diffuse, homogeneous tracer uptake was seen in 101 of 104 reconstructions (97%), correlating with uneventful radiographic and clinical incorporation. In contrast, the 3 hips exhibiting focal “cold” spots—all of which appeared integrated on plain radiographs—progressed to aseptic loosening within 24 months.

Three papers (82 hips) evaluated early implant micromotion as a predictor of long-term graft integration and component survival [9,21,47]. Cups migrating ≤0.3 mm cranially and ≤0.3° in rotation within the first 6 postoperative months (n = 63) stabilized thereafter and have shown no further measurable drift at 5-17 years follow-up. Conversely, every cup with >1 mm early cranial displacement (n = 19)—almost all in large cavitary or segmental defects—failed mechanically at a mean of 3.2 years (range 1-7 years), despite benign radiographic appearances. Low-dose CT-micromotion analysis confirmed this 1 mm “safe zone” and its biological correlate: the single pelvic-discontinuity case that migrated 6 mm also lost 26% of graft mineral density and subsequently loosened.

### Subgroup analysis

#### Lateral cup migration

Notably, only lateral migration was reported in the included studies, with no cases of medial migration identified. Lateral cup migration analysis results are presented in Table 4. In younger patients (under 65), lateral cup migration was greater than those 65 years or older (mean migration: 3.37 mm vs 1.42 mm, *P* = .018). The presence of more severe bone defects (Paprosky Grade III and IV) was associated with higher lateral migration, although this finding did not reach statistical significance (*P* = .135). The subgroup analysis indicated significantly higher lateral migration in cohorts followed for ≥5 years (3.65 mm, 95% CI: −0.27 to 7.57) compared to those with <5 years of follow-up (1.16 mm, 95% CI: −1.08 to 3.41), reaching statistical significance (*P* = .018). This suggests that lateral migration may become more pronounced over time. Although more severe bone defects (often clinically corresponding to higher Paprosky grades) also exhibited numerically greater lateral migration, that finding was not statistically significant in our dataset (*P* = .420).

**Table 4:** Subgroup analysis—lateral cup migration.

Variable examined	Number of studies	Proportion	95% confidence interval	*P* value
Gender				.641
Male	4	2.598	−0.993 to 6.189	
Female	2	2.071	1.174-2.968	
Graft type				.736
Fresh-frozen allograft	3	2.671	−0.207 to 5.550	
Allograft	3	2.133	−4.115 to 8.381	
Antibiotics used				.096
Yes	4	3.050	0.231-5.869	
No	2	1.065	−9.090 to 11.221	
Fixation				.743
Mesh/Screw	2		−10.376 to 16.305	
Rings/Cages	2	3.551	−14.874 to 21.975	
Indication				.420
Bone defect	3	1.794	−3.039 to 6.627	
Aseptic loosening	3	3.009	−1.308 to 7.326	
Age				.135
≥65	3	1.418	−1.056 to 3.892	
<65	3	3.368	−1.667 to 8.402	
Follow-up				.018
≥5 y	3	3.649	−0.273 to 7.570	
<5 y	3	1.162	−1.085 to 3.410	

#### Vertical cup migration

Vertical cup migration did not reveal any significant differences with respect to patient age, graft type, or fixation approach (Table 5). Specifically, in a subgroup analysis comparing ring/cage fixation to mesh/screw fixation, the mean vertical migration for mesh/screw constructs was 3.54 mm (95% CI: 0.41-6.68), whereas ring/cage constructs had a higher mean of 6.10 mm (95% CI: −47.27 to 59.47); however, this difference did not reach statistical significance (*P* = .549). The extremely wide confidence intervals suggest that the sample sizes within each subgroup were likely insufficient to detect meaningful differences—particularly because rings or cages are often reserved for larger bone defects. When stratified by bone loss severity, hips with less severe defects (Paprosky I + II) showed greater vertical migration (mean 4.25 mm, 95% CI: 1.07-7.42) than those with more severe defects (Paprosky III + IV; mean 1.99 mm, 95% CI: 0.73-3.25; *P* < .001).

**Table 5:** Subgroup analysis—superior cup migration.

Variable examined	Number of studies	Mean	95% confidence interval	*P* value
Gender				.333
Male	4	3.132	1.035-5.229	
Female	4	5.246	−1.384 to 11.875	
Graft type				.789
Fresh-frozen allograft	5	3.872	1.320-6.425	
Allograft	3	4.668	−7.485 to 16.820	
Fixation				.549
Mesh/Screw	3	3.543	0.408-6.678	
Rings/Cages	2	6.101	−47.265 to 59.467	
Age				.424
≥65	5	3.373	0.501-6.244	
<65	3	5.503	−5.063 to 16.068	
Follow-up				.335
≥5 y	4	5.226	−0.403 to 10.855	
<5 y	4	3.118	−0.959 to 7.196	
Paprosky				<.001
Grades I + II	2	4.247	1.071-7.423	
Grades III + IV	2	1.988	0.727-3.249

**Table 6:** Subgroup analysis—graft incorporation.

Variable examined	Number of studies	Proportion	95% confidence interval	*P* value
Graft type				.643
Fresh-frozen allograft	8	0.868	0.634-0.961	
Allograft	4	0.986	0.027-1.000	
Autograft	1	1.000	0.000-1.000	
Fixation				.217
Mesh/Screw	6	0.917	0.592-0.988	
Rings/Cages	5	0.995	0.292-1.000	
Age (y)				.392
≥65	7	0.908	0.597-0.985	
<65	6	0.982	0.334-1.000	
Follow-up				.863
≥5 y	10	0.953	0.712-0.994	
<5 y	3	0.934	0.005-1.000	
Paprosky				.125
Grades I + II	2	0.740	0.000-1.000	
Grades III + IV	4	0.955	0.611-1.000	
AAOS				.991
Grade I + II	3	0.931	0.005-1.000	
Grade III + IV	3	0.929	0.006-1.000	

#### Clinical interpretation of pooled migration

Using <3 mm as an acceptable band for nonprogressive displacement on standard radiographs, our pooled lateral mean of 2.4 mm lies within this range, whereas the pooled superior mean of 4.2 mm sits in a 3–5 mm cautionary band where closer surveillance is advisable. Notably, cohorts with <5 years follow-up showed lower lateral migration (1.16 mm) than those with ≥5 years (3.65 mm; *P* = .018), supporting the concept that cranial drift accrues over time. Where high-precision methods were used, early cranial migration ≤0.3 mm within 6 months stabilized long-term, while >1 mm early cranial displacement was uniformly associated with subsequent mechanical failure (n = 19).

#### Additional fixation

Studies were separated into 2 groups: mesh with supplementary-screw group (12 studies, 63%) represents a containment strategy for cavitary defects, whereas the ring/cage group (7 studies, 37%) reflects a bridging approach for segmental loss or pelvic discontinuity (Table 1).

Lateral migration was available from 4 studies (2 mesh/screw [18,20] and 2 ring/cage [6,24] groups). As summarized in Table 4, the effect estimates for the mesh/screw subgroup was 2.9 (95% CI –10.38 to 16.31 mm), while the rings/cages group showed a mean of 3.5 mm (95% CI –14.87 to 21.98; *P* = .743), indicating no detectable difference in early lateral drift.

Vertical migration was reported by 5 studies (3 mesh/screw [6,18,21] and 2 ring/cage groups [24,20]). The mesh/screw cohort migrated a mean of 3.54 mm (95% CI 0.41–6.68), while rings/cages showed 6.10 mm (95% CI –47.27 to 59.47) – however, the difference was not significant (*P* = .549; Table 5).

### Graft incorporation

The subgroup analysis for graft incorporation revealed no major differences between severe and less severe bone loss (95.5% vs 74.0%, *P* = .125, Table 6). Similarly, neither patient age nor the use of additional fixation devices (eg, rings/cages vs mesh/screws) showed a significant influence on graft incorporation. Specifically, graft incorporation rates for patients aged ≥65 years (90.8%, 95% CI: 59.7-98.5) vs those <65 (98.2%, 95% CI: 33.4-100.0) did not differ significantly (*P* = .392). Likewise, rings/cages achieved a 99.5% incorporation rate (95% CI: 29.2-100.0), compared with 91.7% (95% CI: 59.2-98.8) for mesh/screw constructs (*P* = .217). Finally, the choice of fresh-frozen allograft, standard allograft, or autograft did not significantly alter incorporation outcomes (*P* = .643). Together, these findings suggest that graft incorporation is robust across a broad range of defect severities, graft types, patient ages, and fixation methods.

## Discussion

Pooled data show an 89% (95% CI, 79-96%, I^2^ = 85 %) graft-incorporation rate, a mean lateral cup migration average of 2.4 mm (95% CI, 0.53-4.27 mm, I^2^ = 100%), and a mean vertical migration average of 4.18 mm (95% CI, 1.61-6.75 mm, I^2^ = 100 %). While the central tendencies align with prior reports that IBG generates new bone and stable fixation [1,2,30], the very high heterogeneity, nonuniform outcome definitions, and predominantly retrospective designs limit the precision and generalizability of these estimates. Less severe defects (Paprosky I + II) showed greater vertical migration than severe defects (4.25 mm vs 1.99 mm; *P* < .001), perhaps because milder cases were less often reinforced with rings or cages—yielding lower stiffness and more cranial displacement—though inconsistent reporting of fixation by defect grade prevents firm conclusions.

Immediately after impaction, the reconstructed acetabulum is exposed to a vector force generated by the abductors and body weight [48,49] This vector acts craniolaterally and levers the cup from its graft bed—a mechanism commonly termed abduction pull-out. In cadaver and radiostereometric analysis (RSA) studies, the earliest motion is a combination of superior translation and lateral rocking, even when the cup is initially well-seated [9,50]. In our study, we observed the same phenomenon: mean superior cup migration was roughly twice the lateral component (4.2 mm vs 2.4 mm), and cohorts followed ≥5 years demonstrated a progressive increase in lateral displacement.

Abduction pull-out offers a coherent explanation. The impacted chip bed behaves as a noncemented, low-stiffness interface; under cyclic craniolateral load, the superior rim undergoes microcrushing and the cup fulcrums about its inferomedial pole, producing the characteristic cranial-and-lateral migration seen in our meta-analysis. Reinforcement rings or cages do not completely abolish this phenomenon because they still rely on the graft for load transfer [51], but their broader load-spreading flanges may explain the slightly higher (though statistically nonsignificant) superior migration we recorded in the bridging subgroup.

Subgroup analysis revealed that neither preoperative defect severity nor graft type influenced incorporation. Patients with severe bone loss (Paprosky grades III and IV) had incorporation rates comparable to those with less severe defects (95.5% vs 74.0%, *P* = .125). This suggests that IBG is effective even in the presence of extensive bone defects, likely explained by biological capacity of morselized chips to remodel and integrate [52]. Furthermore, patient age, autograft use, and allograft processing (fresh-frozen, γ-irradiated, or freeze-dried) showed no significant influence. Buckley et al. reported 84% incorporation at 5-year with irradiated chips [10], while Ochs et al. observed no outcome between freeze-dried and irradiated grafts [30].

Quantitative imaging supports these clinical findings. Low-dose quantitative CT demonstrated graft density climbing from ∼480 HU immediately postimpaction to 710 HU at 12 months—indistinguishable from adjacent host cancellous bone (720 HU). ^24^Dual-energy X-ray absorptiometry series mirrored this trajectory: Stigbrand et al. recorded a 14% BMD gain by 6 weeks and 23% by 2 years, while Gerhardt et al. reported a 9% increase at 24 months. Together, these data show a steady mineral-accretion pattern without the transient demineralization dip seen in some structural grafts [21,23]. Cup migration is a critical predictor of longevity, as excessive migration is associated with higher failure rates and the need for re-revision surgery [5] Our meta-analysis found pooled mean lateral and vertical migrations of 2.4 mm and 4.18 mm, respectively. Several factors may contribute to the observed heterogeneity. Differences in surgical techniques, fixation methods, patient populations, and follow-up durations can all influence migration. Adjunctive devices are pivotal: studies that added cages or rings reduced vertical drift in cases with severe bone defects [7,8]. Hosny et al. found that a Graft Augmentation Prosthesis (GAP II) cage with IBG achieved stable fixation in high-grade defects, with 100% survivorship at 49 months [6]. Gill et al. likewise reported satisfactory outcomes with a Burch-Schneider cage, noting high incorporation and negligible migration [5]. Patients <65 years exhibited greater lateral migration compared to those aged ≥65 years (mean migration: 3.37 mm vs 1.42 mm, *P* = .018). Greater activity levels expose implants to higher stresses [10], while age-related difference in bone turnover and remodeling may also affect graft integration [52].

For Paprosky IIIA defects with partial rim support, several series and reviews suggest that trabecular metal hemispherical cups with modular augments achieve reliable mid-term fixation and restoration of the hip center, with good radiographic stability and function; these constructs are often preferred when segmental loss precludes chip-only containment but a hemispherical cup can still be supported by augment–host interfaces [53]. In contrast, jumbo cups can be effective for cavitary or contained defects and some IIIA patterns, showing favorable survivorship in systematic and registry reports, but they are less suitable for IIIB or discontinuity where rim support is absent or the hip center would be excessively elevated [54].

For Paprosky IIIB defects and pelvic discontinuity, techniques that bridge the defect are generally recommended over cup-only strategies. Cup–cage constructs show 5-year aseptic-loosening–free survivorship 95% and 85% at 10 years, although all-cause re-revision is higher (80% at 5 years; 68% at 10 years), reflecting the case complexity [55]. Custom triflange acetabular components (CTACs) provide rigid fixation across the ilium–ischium–pubis with patient-specific flanges; contemporary meta-analysis of ∼1200 patients reports effective reconstruction in massive defects (predominantly Paprosky 3A/3B, often with discontinuity), albeit with nontrivial complication and reoperation rates [56]. Narrative reviews similarly position CTACs and cup–cage constructs as leading options when column continuity and rim support are insufficient for cup/augment solutions [57].

In our pooled IBG cohort—largely morselized, nonstructural chips with frequent mesh/screw containment—migration was predominantly cranial and increased with longer follow-up, a pattern consistent with abduction pull-out mechanics. Where segmental loss was larger (typical of IIIB), many authors favored augment with shell, cup–cage, or CTAC to obtain immediate structural stability [58]. IBG is typically adjunctive in these settings (eg, bone chip backfill for augments or inside a cup–cage), reserving chip-only IBG for contained/cavitary defects or when robust containment can be recreated. Practically, for IIIA, IBG (often with containment mesh) or trabecular metal augment–shell reconstructions are both reasonable depending on rim integrity and ability to restore the hip center; for IIIB/discontinuity, cup–cage or CTAC generally provide more predictable stability, with IBG used to restore bone stock behind the load-bearing construct.

Successful IBG depends on creeping substitution [59]: revascularization, osteoclastic resorption, and new host bone replacing the compacted chips [60]. Tagil and Aspenberg showed that impaction accelerates this sequence and strengthens the graft over time, underpinning long-term cup stability and lowering late-loosening risk [61]. Although a postoperative anteroposterior pelvis can look perfectly serviceable, a sizable minority of impaction-graft reconstructions may be nonviable despite normal radiographic presentation. Core biopsies demonstrate that, following impaction bone grafting, morselized cancellous chips serve as an avascular scaffold for the first 2-3 months—held in place solely by the frictional interlock of compaction—and that durable incorporation requires host-vessel ingrowth and ≥70% replacement of the graft by woven bone by 6-12 months; specimens from cups that later failed uniformly retained broad islands of necrotic trabeculae [31,44,52].

This vascular invasion is abrogated by early micromotion, as every cup exceeding 1 mm of early drift—almost all in large cavitary or segmental defects—failed mechanically at a mean of 3.2 years despite deceptively pristine cement–graft interfaces on plain films [9,21,47]. Radionuclide imaging complements this biomechanical data by showing focal cold zones, suggesting low osteoblastic activity in that sector of the graft at 4-6 months (despite normal radiographs)—3 hips that showed these silent areas proceeded to fail aseptically within 24 months [32,44].

As a summary for hypothesis-generating clinical decision making: for contained/cavitary defects (Paprosky I–II and selected IIIA with recreated containment), chip-only IBG remains a bone-stock–restoring option; study-level data show no efficacy difference between autograft and (fresh-frozen or irradiated) allograft, choice can be made based on availability and infection control. For IIIB or pelvic discontinuity, IBG may be used adjunctively (as backfill) with load-bearing constructs (augment–shell, cup–cage, custom triflange) to secure immediate stability. Because migration is predominantly cranial and may accrue over time, we recommend standardized radiographic surveillance (baseline, 6 and 12 months, then annually) and apply thresholds: <3 mm nonprogressive = acceptable; 3–5 mm = caution/closer follow-up; progressive ≥5 mm and/or >5° inclination = concerning; >1 mm early cranial drift on RSA/CT at 6–24 months warrants closer review. Given most source data involve cemented cups, guidelines can reasonably prefer cemented fixation over a well-compacted graft when containment is adequate, reserving uncemented cups for robust host–bone contact with adjunctive fixation.

This meta-analysis has several limitations. Most included cohorts were retrospective, nonrandomized, and of moderate methodological quality (MINORS), so selection bias and unmeasured confounding (activity level, bone quality, surgeon/center effects) cannot be excluded. Outcome definitions and measurement methods varied: “graft incorporation” was reported using differing radiographic criteria, and migration was assessed with plain radiographs vs CT/CT-based micromotion analysis (occasionally RSA), at nonuniform time points and thresholds—factors that inflate heterogeneity and limit direct comparisons. Denominators therefore differ by outcome, hip-level stratification by Paprosky grade, fixation adjuncts, or graft material was uncommon—as a result, key contrasts (eg, structural vs nonstructural IBG, cemented vs uncemented, autograft vs allograft) were underpowered or not feasible beyond study-level subgrouping. Finally, small-study/publication bias signals were observed for some outcomes, and several subgroup contrasts (eg, fixation type by defect grade, cemented vs uncemented cups) were insufficiently powered—causal inferences should be avoided.

## Conclusions

Acetabular IBG provides favorable mid- to long-term overall graft incorporation rate, averaging 89% with significant data heterogeneity (I^2^ = 85%). Neither the amount of preoperative bone loss, graft type, additional fixation or patient age significantly impacted graft incorporation. Cup migration could be observed at mid-term follow-up, mostly within acceptable thresholds.

## Conflicts of interest

The authors declare there are no conflicts of interest.

For full disclosure statements refer to https://doi.org/10.1016/j.artd.2025.101929.

## CRediT authorship contribution statement

**Artsiom Klimko:** Writing – review & editing, Writing – original draft, Methodology, Investigation, Formal analysis, Data curation, Conceptualization. **Octavian Andronic:** Writing – original draft, Supervision, Project administration, Methodology, Investigation, Conceptualization. **Victor Yan Zhe Lu:** Writing – original draft, Software, Resources, Investigation, Formal analysis. **Dominik Kaiser:** Writing – original draft, Visualization, Supervision, Resources, Project administration, Methodology. **Dimitris Dimitriou:** Writing – original draft, Project administration, Methodology, Investigation, Funding acquisition, Formal analysis, Data curation, Conceptualization. **Armando Hoch:** Writing – original draft, Supervision, Resources, Methodology, Investigation, Funding acquisition, Formal analysis, Data curation. **Patrick O. Zingg:** Writing – review & editing, Writing – original draft, Visualization, Validation, Supervision, Formal analysis, Data curation.

## Appendix A. Supplementary data

Supplementary data related to this article can be found at https://doi.org/10.1016/j.artd.2025.101929.

## Appendix A Supplementary data

Conflict of Interest Statement for Klimko

Conflict of Interest Statement for Andronic

Conflict of Interest Statement for Lu

Conflict of Interest Statement for Dimitriou

Conflict of Interest Statement for Hoch

Conflict of Interest Statement for Zingg

Conflict of Interest Statement for Kaiser
