# Supplementary material for: Graft Incorporation and Cup Migration in Acetabular Impaction Bone Grafting for Revision Hip Arthroplasty: A Systematic Review and Meta-Analysis of 1093 Hips
Source: Arthroplast Today. 2026 Jan 14;37:101929. doi: 10.1016/j.artd.2025.101929 (PMC12828803; doi:10.1016/j.artd.2025.101929)
Supplement: Conflict of Interest Statement for Lu [file mmc3.docx]

# INDIVIDUAL CONFLICT OF INTEREST STATEMENT

***American Association of Hip and Knee Surgeons***

(Adopted from the American Academy of Orthopaedic Surgeons disclosure statement)

The following form **must be filled out completely and submitted by each author (example, 6 authors, 6 forms).**

**All items require a response. If there is no relevant disclosure for a given item, enter "*None*.”**

**Manuscript Title**

" Graft Incorporation and Cup Migration in Acetabular Impaction Bone Grafting for Revision Hip Arthroplasty: A Systematic Review and Meta-Analysis of 1,093 hips "

This conflict of interest form is for author Victor Yan Zhe Lu, MA MB BChir

2 - Department of Orthopaedics and Traumatology, The Chinese University of Hong Kong, China

1. Royalties from a company or supplier (The following conflicts were disclosed)

None

2. Speakers bureau/paid presentations for a company or supplier (The following conflicts were disclosed)

None

3A. Paid employee for a company or supplier (The following conflicts were disclosed)

None

3B. Paid consultant for a company or supplier (The following conflicts were disclosed)

None

3C. Unpaid consultants for a company or supplier (The following conflicts were disclosed)

None

4. Stock or stock options in a company or supplier (The following conflicts were disclosed)

None

5. Research support from a company or supplier as a Principal Investigator (The following conflicts were disclosed)

None

6. Other financial or material support from a company or supplier (The following conflicts were disclosed)

None

7. Royalties, financial or material support from publishers (The following conflicts were disclosed)

None

8. Medical/Orthopaedic publications editorial/governing board (The following conflicts were disclosed)

None

9. Board member/committee appointments for a society (The following conflicts were disclosed)

None

**Each author must sign AND print or type his/her name, date and submit a separate form**

In addition, one BLINDED Conflict of Interest form (no author names used) should be submitted per manuscript with all author disclosures.

Victor Yan Zhe Lu VL January 29^th^, 2025

Author Name (Print or Type) Author Signature Date
